# Endoscopic Closure of a Spontaneous Esophageal Perforation Using an Over-the-Scope Clip (OTSC®): A Conservative Approach to Boerhaave Syndrome

**DOI:** 10.7759/cureus.83442

**Published:** 2025-05-04

**Authors:** Eduarda Magalhães, Mariana Fonte Martins, Margarida Fertusinhos, Hugo Palma Rios, Dina Luís

**Affiliations:** 1 General Surgery, Unidade Local de Saúde de Braga, Braga, PRT; 2 Radiology, Unidade Local de Saúde de Braga, Braga, PRT; 3 Surgical Gastroenterology, Unidade Local de Saúde de Braga, Braga, PRT

**Keywords:** boerhaave syndrome, conservative management, endoscopic therapy, oesophageal perforation, otsc

## Abstract

Boerhaave syndrome (BS), a rare and potentially fatal condition characterized by the spontaneous transmural rupture of the esophagus, often poses an initial diagnostic challenge due to its nonspecific clinical manifestations. Contrast-enhanced computed tomography (CT) is the preferred imaging modality for diagnosis. We present the case of a 63-year-old patient with multiple comorbidities who developed acute retrosternal chest pain following an episode of vomiting. Radiological imaging demonstrated a distal esophageal perforation, leading to the implementation of a conservative treatment approach involving ventilatory support, empirical antibiotic therapy, and thoracic drainage. Upper endoscopic examination identified the perforation, which was effectively closed with an Over-the-Scope Clip (OTSC^®^; Ovesco Endoscopy AG, Tübingen, Germany). The patient experienced a positive clinical course, with resolution of the pneumothorax and subsequent transfer to a rehabilitation center. This case highlights the necessity of individualized therapeutic strategies in the management of BS and demonstrates the potential of minimally invasive endoscopic techniques, such as OTSC^®^, as an effective option in stable and appropriately assessed patients.

## Introduction

Boerhaave syndrome (BS) is a rare and potentially fatal condition characterized by the spontaneous rupture of the esophageal wall, typically resulting from a sudden increase in intraesophageal pressure, often associated with episodes of vomiting [1]. Common symptoms include severe chest pain, dysphagia, vomiting, and, in some cases, signs of septicemia. However, these symptoms occur in only 14% of cases, according to the literature [2]. Diagnosis is often confirmed through imaging studies, such as radiographs or computed tomography (CT), which may reveal air in the thoracic cavity indicative of the rupture [3].

This condition, first described by Herman Boerhaave in the 18th century, presents as a surgical emergency that, if left untreated, can progress to mediastinitis, septic shock, and other severe clinical complications [2,4]. Timely diagnosis and treatment are crucial for improving patient prognosis. However, not all cases require immediate surgical intervention. In selected patients, conservative management - including supportive care and antibiotic therapy - may be a viable alternative [5,6].

This case report details the clinical presentation, treatment, and evolution of a 63-year-old male patient with several comorbidities, who was admitted to Hospital de Braga due to BS, with a focus on the decision to adopt a conservative therapeutic approach. This case highlights the challenges in managing such a condition, particularly in the face of complications such as mediastinitis, pneumothorax, and other intercurrent issues.

## Case presentation

The male patient had a significant medical history, including obesity, chronic obstructive pulmonary disease (COPD), hypertension (HTN), bilateral chronic otitis media (COM) with previous mastoidectomy, a history of childhood craniotomies (etiology uncertain), a history of smoking (former smoker), and alcohol consumption (reported as decreased). Admission occurred on October 4, 2024, at Barcelos Hospital due to sudden chest and abdominal pain, with approximately one hour of progression, associated with dyspnea and episodes of diarrhea. The patient also reported a history of vomiting on the same day, preceding the onset of continuous retrosternal pain, assessed at an intensity of 10/10. The initial suspicion at Barcelos Hospital was acute coronary syndrome, which was subsequently ruled out.

On initial physical examination at Barcelos Hospital, the patient presented as polypneic, with an oxygen saturation (SpO_2_) of 96% on FiO_2_ 0.40, heart rate (HR) of 120-140 bpm, and blood pressure (BP) of 155/97 mmHg. The abdomen was distended and tender to palpation in the epigastric region, without clear signs of peritoneal irritation. After placement of a nasogastric tube (NGT), 500 mL of brownish content was drained.

Complementary examinations began with analyses performed at Barcelos Hospital prior to transfer. The complete blood count showed leukocytosis (14.90 × 10³/mm³), with 81.90% neutrophils and 11.90% lymphocytes. Platelets were elevated (419 × 10³/mm³). Biochemical analyses revealed glucose at 210 mg/dL, urea at 46 mg/dL, creatinine at 1.11 mg/dL, sodium at 141 mmol/L, potassium at 4.2 mmol/L, and chloride at 103 mmol/L. Liver function assessment showed normal values for aspartate aminotransferase (AST), alanine aminotransferase (ALT), gamma-glutamyl transferase (GGT), total amylase, and lipase.

Troponin and myoglobin values were within reference parameters, demonstrating the absence of myocardial injury. Prothrombin time and activated partial thromboplastin time (aPTT) values were within reference limits (International Normalized Ratio, or INR 0.97 and aPTT 21 seconds). C-reactive protein (CRP) values were below 0.50 mg/dL. The detailed presentation of these results, including reference values, is found in Table 1.

**Table 1:** Results of analyses performed at Hospital de Barcelos CRP, C-reactive Protein; INR, International Normalized Ratio; aPTT, Activated Partial Thromboplastin Time; AST, Aspartate Aminotransferase; ALT, Alanine Aminotransferase; GGT, Gamma-Glutamyl Transferase

Parameter	Result (With Unit)	Reference Range
Complete Blood Count		
Leukocytes	14.90 × 10³/mm³	4.50 - 11.00
Neutrophils	81.90%	40.0 - 60.0
Lymphocytes	11.90%	20.0 - 40.0
Platelets	419 × 10³/mm³	150.0 - 400.0
Biochemistry		
Glucose	210 mg/dL	60 - 110
Urea	46 mg/dL	<50
Creatinine	1.11 mg/dL	0.70 - 1.20
Sodium	141 mmol/L	135 - 145
Potassium	4.2 mmol/L	3.5 - 5.5
Chloride	103 mmol/L	96 - 115
Coagulation		
INR	0.97	0.8 - 1.2
aPTT	21 seconds	26 - 36
Other Parameters		
CRP	<0.50 mg/dL	<0.8
AST, ALT, GGT, Total Amylase, Lipase, Troponin, Myoglobin	Within reference values	-

**Figure 1:**
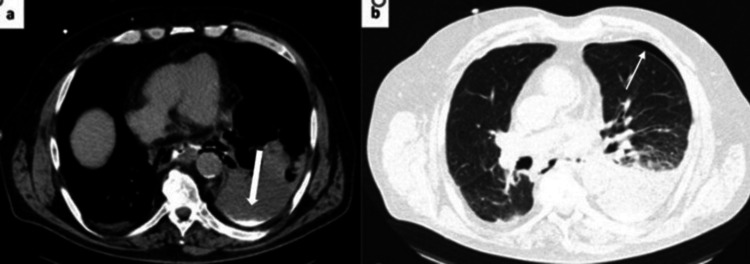
(a) CT scan with oral contrast, which demonstrates in the topography of the lower third of the esophagus, leakage of contrast material into the left pleural space, associated with ipsilateral pleural effusion, in relation to esophageal rupture (thick arrow); (b) Presence of a thin lamella of pneumothorax on the left (thin arrow). CT, Computed Tomography

CT of the thorax, abdomen, and pelvis, performed on October 3, 2024, at Hospital de Barcelos, revealed a small left-sided pneumothorax, a small lower pneumomediastinum, and a moderate left-sided pleural effusion, confirming the suspected esophageal perforation in the lower third (Figure 1).

Empiric antibiotic therapy with ceftriaxone and metronidazole was initiated in Barcelos. After evaluation by General Surgery, the patient was transferred to Hospital de Braga under the care of the surgical team and admitted to the Polyvalent Intensive Care Unit (UCIP). In the UCIP, a proton pump inhibitor (PPI) infusion (8 mg/h) was initiated, and a chest tube was inserted into the left hemithorax, draining 800 mL of brownish fluid that was collected for microbiological analysis. Upon admission to the UCIP, the patient was alert, cooperative, oriented, and reported thoracic discomfort, with polypnea (SpO_2_ 92% and FiO_2_ 0.31) and an oscillating, bubbling chest tube. A right radial artery catheter and a left internal jugular central venous catheter were inserted, and therapy was adjusted.

In the UCIP, the patient was evaluated by Gastroenterology on 7/10. An upper endoscopy revealed, approximately 3 cm from the gastroesophageal junction (GEJ), a small discontinuity, with a more distal laceration extending up to the apparently closed GEJ. An Over-the-Scope Clip (OTSC^®^; Ovesco Endoscopy AG, Tübingen, Germany) was placed to encompass the discontinuity - apparently with success.

**Figure 2:**
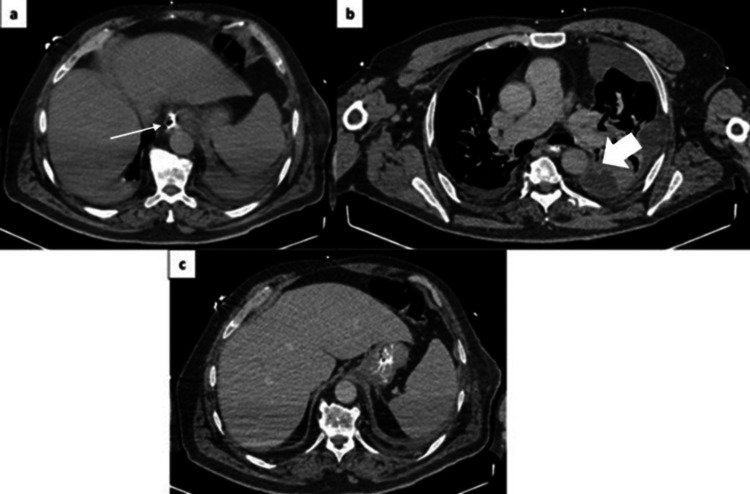
(a) Non-contrast CT showing the presence of hyperdense material in the region of the esophageal rupture, related to the conservative treatment performed by endoscopy (thin arrow); (b) and (c) After administration of oral contrast, passage of contrast into the gastric cavity is noted without evidence of leakage. Additionally, persistence of the bilateral pleural effusion, partially loculated on the left, is seen (thick arrow). CT, Computed Tomography

Radiological assessment with a CT scan of the thorax, abdomen, and pelvis on October 11, 2024 (Figure 2), showed resolution of the pneumothorax but demonstrated pneumoatelectatic densities in the lower lobes and lingula; paraseptal and centrilobular emphysema (more evident in the upper lobes); a small bilateral pleural effusion (partially loculated on the left, with small gas bubbles); and slight emphysema of the homolateral thoracic wall (related to the insertion of the chest tube).

Analysis of the pleural fluid confirmed mediastinitis, with isolation of *Candida glabrata*, *Candida tropicalis*, *Staphylococcus aureus*, and *Lactobacillus rhamnosus*. Antifungal treatment with fluconazole was initiated, and antibiotic therapy was continued. Despite favorable progress, the patient continued to require oxygen therapy and experienced difficulty with ventilator weaning.

After 15 days in the UCIP, the patient demonstrated clinical improvement and was transferred to the surgery ward, where he initiated a diet with good tolerance, maintained oxygen therapy via nasal cannula at 2 L, and began physical therapy due to motor weakness. At discharge, the patient was improved but still required oxygen via nasal cannula at 2 L and continued physical therapy for motor weakness. The post-discharge care plan included transfer to a rehabilitation hospital in his area of residence, where he remained hospitalized for another week, with successful weaning from oxygen therapy and subsequent discharge home.

## Discussion

BS is a rare but highly lethal condition characterized by spontaneous transmural rupture of the esophagus, typically triggered by forceful vomiting or a sudden increase in intraesophageal pressure against a closed glottis [1].

Since its first description by Hermann Boerhaave in 1724, BS remains a significant diagnostic and therapeutic challenge, with mortality rates ranging from 30% to 50%, potentially reaching 100% without timely and appropriate intervention [2,3]. Ruptures most commonly occur in the lower third of the esophagus, typically on the left posterolateral wall, approximately 2-4 cm above the GEJ [7].

The present case illustrates the diagnostic complexity of BS, given its often nonspecific clinical presentation. Although Mackler’s triad - vomiting, chest pain, and subcutaneous emphysema - is considered characteristic, it is observed in only a minority of cases [8,9]. In this case, the patient presented with severe retrosternal chest pain without subcutaneous emphysema, which contributed to an initial diagnostic delay. This highlights the need for a high index of clinical suspicion, as early diagnosis is directly correlated with improved outcomes.

A chest CT scan performed shortly after admission played a pivotal role in diagnosis, revealing pneumomediastinum and pleural effusion - findings consistent with esophageal rupture. This is in line with existing literature, which supports contrast-enhanced CT as the imaging modality of choice for BS, with a reported sensitivity of 90%-100% [10]. In this case, an additional contrast esophagogram further delineated the extent of the perforation and guided therapeutic planning.

Management options for spontaneous esophageal perforation include endoscopic stenting, primary suture repair (with or without reinforcement), double esophageal exclusion, and esophagectomy [11,12]. It is well documented that survival rates decrease significantly when diagnosis is delayed beyond 24 hours [13].

Surgical management is generally recommended for septic patients with esophageal perforation, whereas conservative treatment is typically reserved for those presenting early and without sepsis. In selected cases of delayed presentation without systemic infection, non-operative management may also be appropriate. Recovery in such patients may be further accelerated through minimally invasive endoscopic techniques [14,15]. In the present case, a conservative strategy was chosen, comprising ventilatory support, empirical antimicrobial therapy, and thoracic drainage. Endoscopic placement of the OTSC^®^ was instrumental in sealing the perforation and preventing further leakage.

The literature supports that conservative management should be reserved for well-selected patients who meet strict criteria [11-17]. In a meta-analysis encompassing 75 studies on esophageal perforations of various etiologies, Biancari et al. concluded that the choice of management strategy is largely influenced by the individual surgeon’s experience and preference, given the absence of robust evidence favoring one specific approach over another [18]. 

Matsuda et al. [19] reported the case of a 41-year-old male patient presenting with hematemesis and severe epigastric pain. The initial suspicion of gastrointestinal tract perforation - specifically a perforated peptic ulcer - led to the performance of an upper gastrointestinal endoscopy, which established the diagnosis of BS. The authors opted for a conservative approach, based on the patient’s hemodynamic stability, absence of sepsis, early diagnosis, and a contained esophageal laceration. The conservative management proceeded without complications and resulted in a favorable outcome.

Regarding the antibiotic regimens reported in the literature for conservatively managed cases, all involve broad-spectrum antibiotics. Matsuda et al. [19] described the use of imipenem; Anwuzia-Iwegbu et al. [20] employed piperacillin/tazobactam (Tazocin) in combination with metronidazole. In the present case, the patient was treated with ceftriaxone - a third-generation cephalosporin with good Gram-positive and excellent Gram-negative coverage - alongside metronidazole, a nitroimidazole-class bactericidal antibiotic effective against anaerobes and protozoa. Following the microbiological results of the pleural fluid, it was necessary to add fluconazole to the previously established antibiotic regimen, which proved effective in this patient.

The patient’s clinical course underscores the importance of a multidisciplinary approach involving surgery, intensive care, and gastroenterology teams. Supportive measures, such as parenteral nutrition and an adequate period of fasting, were fundamental for esophageal healing. Despite the initial severity, the patient showed gradual clinical improvement, with resolution of the pneumothorax and successful discharge from the hospital.

Functional recovery after esophageal perforation varies and depends on multiple factors, including the extent of the injury, baseline nutritional status, and complications such as mediastinitis or ventilator-associated pneumonia. In this case, the patient required prolonged oxygen therapy and motor rehabilitation, consistent with reports in the literature describing ventilatory weaning difficulties in patients with significant comorbidities.

In conclusion, this case reinforces the critical importance of early diagnosis and an individualized therapeutic strategy in the management of BS. Successful conservative treatment hinges on strict patient selection and rigorous clinical monitoring to detect early signs of deterioration. This case adds to the growing body of evidence supporting conservative and endoscopic approaches as feasible options in selected clinical scenarios.

## Conclusions

BS, while a rare and life-threatening condition, necessitates prompt recognition and an individualized therapeutic strategy. This case demonstrates that, in carefully selected patients exhibiting contained esophageal perforation, clinical stability, and the absence of severe sepsis, conservative management - including endoscopic closure utilizing the OTSC^®^ - can present a safe and efficacious alternative to surgical intervention. The favorable clinical outcome underscores the importance of a multidisciplinary approach, rigorous monitoring for complications such as mediastinitis, and the burgeoning role of minimally invasive endoscopic techniques in the management of esophageal perforations. This case contributes to the expanding body of evidence supporting conservative treatment in appropriately selected patients.
